# The association between urinary lgM excretion and diabetic retinopathy in diabetic patients

**DOI:** 10.1186/s40200-016-0242-x

**Published:** 2016-06-29

**Authors:** Mojgan Sanjari, Gholam Reza Asadikaram, Fariba Beigzadeh, Soheila Torabian, Zohreh Safi, Amirfarhad Ghaseminejad Tafreshi

**Affiliations:** 1 Endocrinology and metabolism Research Center, Institute of Basic and clinical physiology Sciences, Kerman University of Medical Sciences, Kerman, Iran; 2 Physiology Research Center, Institute of Neuropharmacology, Kerman University of Medical Sciences, Kerman, Iran; 3 Kerman University of Medical Sciences, Kerman, Iran; 4 University of British Columbia (UBC), 2329 West Mall, Vancouver, BC V6T 1Z4 Canada

**Keywords:** Diabetes Complications, Diabetic Retinopathy, Urinary IgM

## Abstract

**Background:**

Diabetic Retinopathy is one of the most common causes of blindness among adults. Microvascular complications may have common origins. The objective of the present study is to analyze the correlation between urinary IgM excretion and diabetic retinopathy based on the type of diabetes.

**Methods:**

The present study is cross-sectional analytic and was carried out on 140 type2 diabetic patients (of which 70 patients diagnosed with retinopathy) and 76 type1 diabetic patients (of which 37 patients diagnosed with retinopathy). For every patient in each of the test groups, fasting plasma glucose, triglyceride, cholesterol, creatinin and HbA1c tests were done. The value of IgM, the albumin- to- creatinine ratio and the urine analysis test were also used to rule out the significant proteinuria of the patients. Then, IgM Index was measured using the following equation: Igm Index = Urine IgM/Urine Cr.

**Results:**

The level of IgM index in the diabetic patients (type1 and type2) had no significant correlation with retinopathy. Cut point = 1.49, sensitivity = 0.703 and specificity = 0.308 in type1 diabetes were used for screen retinopathy. In type1 diabetic patients, the duration of diabetes had a significant correlation with urinary protein while in type 2 diabetic patients, the diabetes duration and HbA1c were significantly correlated with retinopathy.

**Conclusion:**

The results of this study demonstrate that the level of urinary IgM in diabetic patients has no difference in those who have or lack retinopathy, but the urinary IgM level of more than 1.49 mg/dl can be considered as a cut point in type1 diabetic patients to screen retinopathy.

## Background

Diabetes mellitus includes a group of common metabolic disorders among which the distinguishing characteristic is phenotypic hyperglycemia. The global incidence of diabetes mellitus increased from 30 million cases in 1985 to 285 million in 2010. In Kerman- Iran, the standardized prevalence of diabetes was 9 % (men 7.7 % and women 10.3 %) [1]. Moreover, in Iran as a developing country, about 6 % of the population suffers from type2 diabetes and its complications. As a result, the morbidity and mortality of this disease is also increasing [2, 3].

Diabetic retinopathy is one of the microvascular complications of diabetes and is also one of the most common reasons for blindness among 20 to 74 year-old adults [4, 5]. The type and duration of diabetes, age, sex, glycemic control, systemic hypertension,body mass index, smoking, and serum lipid and microalbuminuria are all associated with the progress of diabetic retinopathy [6–10].

Microalbuminuria is significantly correlated with diabetic retinopathy in type2 diabetes, and is therefore a reliable marker for retinopathy [11]. In type2 patients, the increase of urinary excretion of albumin is significantly correlated with retinopathy, neuropathy and cardiovascular diseases [12].

The transfer of proteins from capillary walls of glomeruli is done based on the size and electrical load of the molecules. One could say that the walls of glomeruli have the ability of transferring molecules based on the size and charge of macromolecules [12–14]. In a model with two pores and one shunt [15], a pore with a small diameter and radius of 2.9-3.1 nanometers is supposed for the transfer of negatively charged globular proteins and a small number of pores with larger radiuses (8–9 nanometers) are supposed for the transition of larger proteins [16].

In normal conditions, smaller pores are impenetrable for the transfer of albumin which has a radius of 3.6 nanometers [17]. However, in case there is a lack of a negative charge, proteins with the radius of 4.5- nanometers can pass through such pores [16–18]. Larger proteins with a radius of higher than 5.5 nanometers such as IgM cannot pass through normal pores and do not need shunts of glomerular wall [16–18]. Therefore, it seems that the mechanisms of albumin excretion and IgM excretion in kidney are different and on the other hand, it appears that these mechanisms are different in type1 and type2 diabetes. In patients with type2 diabetes, urinary IgM excretion is more than patients with type1 diabetes. Moreover, in 86 % of type1 and 35 % of type2 diabetic patients, urinary concentration of IgM was undetectable [19]. On the other hand, the association between vascular complications and diabetes is completely known. In some studies, the significant correlation between vascular complications and urinary IgM excretion has been supported in a way that urinary IgM excretion increases the possibility of mortality due to cardiovascular complications and renal disorders in patients with type1 diabetes without considering the degree of albuminuria [20]. It is also observed that in type2 diabetic nephropathy, the increase of urinary IgM is followed by mortality due to cardiovascular and renal events [21].

The correlation of urinary IgM excretion with the progress of renal disorder and mortality of cardiovascular events has been verified. Though, such correlation has not been investigated for retinopathy. Micro-vascular complications might have common origins. Therefore, it seems that urinary IgM excretion may be significantly correlated with retinopathy. In the present study, the objective is to verify the correlation between urinary IgM excretion and diabetic retinopathy based on the type of diabetes.

## Methods

The present study is cross-sectional and analytical. The control group included 216 diabetic patients of whom 140 patients had type2 diabetes and 76 patients had type1 diabetes. Among the 140 type2 patients, 70 had retinopathy while there were 37 cases of retinopathy within the 76 patients with type1 diabetes. These patients were chosen from the visitor of clinical division of endocrinology in Afzalipur Hospital of Kerman city (center of a province in southern Iran).

The inclusion criteria were type1 and type2 diabetes. The diagnosis of diabetes was done based on the standards of ADA 2014 and confirmed by endocrinologists. In order to diagnose type1 diabetes, standards of less than 30 year-old were applied which included acute onset and diabetic ketoacidosis. If the patients were older than 30 years, less-than-one level of c-peptide (c-peptide ≤ 1 ng/ml) was applied as the inclusion criteria for type1. In the information form the duration of diabetes, age, sex, level of glycemic control, blood pressure, body mass index, smoking and serum lipid levels were highlighted and adjusted in the statistical calculations.

The exclusion criteria were significant proteinuria (macro-albuminuria in urine), creatinin > 1.5 mg/dl and GFR < 90 cc/min per 1.73 m^2^.

After confirmation through diagnosis and initial inspection, the included patients filled the initial forms and the agreement forms to participate in the study. They were also given two information papers to visit an ophthalmologist and get tested at a laboratory. In the laboratory, 5 cc (mL) clotted blood of each individual was sampled to do fasting plasma glucose, triglyceride, cholesterol and creatinin tests. 1 cc (mL) of patients’ blood was also used to do HbA1C tests. All of the mentioned tests were done through the calorimetric method. HbA1c was measured through Nycocard/reader П and Nycocard kit and its normal value was considered to be less than 6.5 % [4]. In addition, a random sample of urine was used to measure the value of IgM and albumin-to-creatinin ratio. Urine analysis test was used to rule out the patients with significant proteinuria. Urinary albumin was measured through torbidometeric method by Perestige system and Randox kit; considering that the normal value of urinary albumin is less than 20. Urinary IgM was also measured using the colorimetric method and by Eliza reader and Bethyl kit. At the end, the IgM index was calculated using the following formula [19]:

$$ \mathrm{I}\mathrm{g}\mathrm{m}\ \mathrm{I}\mathrm{ndex} = \mathrm{Urine}\ \mathrm{I}\mathrm{g}\mathrm{M}\ /\ \mathrm{Urine}\ \mathrm{C}\mathrm{r} $$

1.8 × 10 ^-3^ was regarded as the cut point of IgM index for type1 diabetes. Then, all of the patients had eye examination with open pupils using an ophthalmoscope done by an ophthalmologist to determine the existence of retinopathy and its stage.

Finally, the collected data were analyzed through SPSS Software (version. 21), the tests of analytical and descriptive statistics such as T test،X^2^, regression and linear multivariate along with univariate tests. Subsequently, Roc curve was drawn for both groups.

## Results

(Table 1) Based on the information, the mean age in type1 diabetic patients with retinopathy is more than those diabetic patients without retinopathy. Also the mean age in type2 diabetic patients with retinopathy is more than those without retinopathy. Furthermore, the gender of patients who suffer or do not suffer from retinopathy do not show any difference in the type of diabetes. The duration of diabetes is longer in type1 diabetic patients with retinopathy. Likewise, the duration of diabetes is longer in type 2 diabetic patients with retinopathy. In individuals who suffer from retinopathy, hypertension is more common in both types of diabetes with the) *p* ≤ 0.047) in diabetes type1 and (*p* ≤ 0.003) in diabetes type2.

**Table 1:** Shows demographic and laboratory information of diabetic patients with type1 and type 2

Variable	DM type I			DM type II		
	Retinopathy Yes, *N* = 37	Retinopathy No, *N* = 39	*P*-value	Retinopathy Yes, *N* = 70	Retinopathy No, *N* = 70	*P*-value
Age (year)	34.1	23.6	<0.001	49.8	45.6	<0.001
Sex (female)	17	24	0.170	56 (80 %)	49 (70 %)	0.170
Duration (year)	15.8	7.6	<0.001	12.5	7.0	<0.001
HTN (yes)	9 (24.3 %)	3 (77 %)	0.047	36 (51.4)	19 (27 %)	0.003
Mean arterial BP	81/9 ± 8/0	87/5 ± 8/4	0/004	91/6 ± 15/7	90/7 ± 10/3	p = 0/6
Systolic BP (mm Hg)	110/6 ± 11/98	119/3 ± 15/5	<0/05	118/6 ± 14/6	133/109 ± 1/8	P = 0/0001
Diastolic BP (mm Hg)	67/6 ± 7/1	71/6 ± 6/4	<0/01	72/2 ± 9/1	75/1 + 8/6	P = %6
BMI (kg/m2)	25.1	22.5	0.015	30.0	29.8	0.773
Cigarette (yes)	6 (16.2 %)	2 (5.1 %)	0.148	9 (12.9 %)	2 (2.9 %)	0.028
FPG (mg/dl)	162.4	172.7	0.650	162.7	138.18	0.045
HbA1c%	7.7	7.7	0.920	8.2	7.1	<0.001
TRIGLYCERIDE (mg/dl)	154.5	133.3	0.287	190.2	193.4	0.870
Cholesterol (mg/dl)	191.6	173.0	0.068	186.0	184.5	0.784
Urine Albumin (mg/dl)	30.5	20.3	0.074	30.6	20.3	0.063
Urine Creatinine (mg/dl)	198.5	141.0	0.132	137.3	223.9	<0.001
Urine protein (mg/l)	222.7	104.3	<0.001	15.4	15.4	0.900
IgM (mg/dl)	0.114	0.096	0.530	0.191	0.044	0.167
IgM Index	6.2	4.1	0.192	15.6	12.7	0.565

Mean arterial blood pressure was higher in patients who suffer from retinopathy. (92/1 ± 9/8 V S 85/6 ± 9/8, *P* = 0/0001) One third of patients were receiving antihypertensive medication including losartan (*N* = 50, 23 %) enalapril (*N* = 6), captopril (*N* = 2), amlodipin (*N* = 12) and atenolol (*N* = 8). Since treatment with antihypertensive might interfere in the interpretation of results related to Igm, the level of Igm was compared in patients with and without medication use that less to no significant differences. Additionally, in type1 diabetic patients with retinopathy, the mean BMI is significantly higher. However, this difference is not observed in type2 diabetes.

The results also indicate that smoking is not correlated with retinopathy in either types of diabetes.

The mean FPG in type2 diabetic patients with retinopathy is significantly more than those who lack retinopathy (162.78 ± 78.6 versus 138.18 ± 64.54) with the *p*-value of (0.045). Though, this difference is not observed in type1 diabetes.

The mean HbA1C in type2 diabetic patients with retinopathy is significantly more than those who lack retinopathy (8.2 ± 1.69 versus 7.1 ± 1.67) with the *p*-value of (0.001). However, this difference is not observed in type1 diabetes.

Neither types of diabetes have significant differences in their levels of Triglyceride, cholesterol or albumin between the two groups regardless of having diabetic retinopathy.

The mean urinary protein in type1 diabetic patients with retinopathy is significantly more than those who lack retinopathy (22.27 ± 11.42 versus 10.43 ± 10.48) with the *p*-value of (0.001), but the same is not the case for type2 diabetes.

Neither types of diabetes have significant differences in their IgM index value between the two groups regardless of having diabetic retinopathy.

(Table 2) Based on univariate linear regression analysis Table 2, in type1 diabetic patients, the correlation of age, duration of diabetes, HTN, BMI and urinary protein with retinopathy is significant. Moreover a significant correlation between retinopathy and sex, smoking, FPG, HbA1c, Triglyceride, Cholesterol, micro-albuminuria, Urine IgM, Urine IgM index and urine protein is supported.

**Table 2:** Correlation of All Variables with IgM in Type I and II Diabetic Patients (Univariate)

Variable	DM type I		DM type II	
	OR CI (95 %)	*P*-value	OR CI (95 %)	*P* value
Age	1.12 (1.05–1.18)	<0.001	1.16 (1.08–1025)	0.001
Sex (Female)	0.53 (0.21–1.32)	0.16	1.71 (0.78–3.7)	0.17
Duration	1.21 (1.10–1.32)	0.001	1.22 (1.12–1.32)	0.001
HTN	3.8 (0.95–15.5)	0.058	2.8 (1.4–5.7)	0.04
BMI	1.14 (1.02–1.27)	0.02	1.012 (0.193–1.09)	0.77
Cigarette	3.5 (0.67–19.00)	0.13	5.01 (1.04–24)	0.044
FPG	0.99 (0.99–1.00)	0.65	1.005 (1–1.01)	0.05
HbA1c	1.01 (0.18–1.26)	0.92	1.49 (1.19–1.86)	0.001
Triglyceride	1.06 (0.33–3.4)	0.91	1.46 (0.72–2.9)	0.28
Cholestrol	1.37 (0.152–3.630)	0.51	1.37 (0.67–2.7)	0.37
Urine Albumin	1.13 (0.198–1.3)	0.078	1.09 (0.99–1.19)	0.07
Urine Creatinine	1.03 (0.999–1.007)	0.16	0.995 (0.992–0.998)	0.001
Microalbuminuria	2.02 (0.74–21.3)	0.16	1.34 (0.68–2.62)	0.39
Urine Protein	7.2 (2.4–21.3)	0.001	1.23 (0.59–2.55)	0.57
IgM Index	1.05 (0.97–1.13)	0.20	1.003 (0.992–1.015)	0.57
IgM	4.6 (0.49–43.2)	0.18	1.72 (0.74–4.01)	0.20

The results of this test indicate that there is no significant correlation between retinopathy and measured factors such as age, duration of diabetes, HTN, smoking, FPG, HbA1c and Creatinin in type2 diabetic patients. The same is the case for the relationship between sex, BMI, cholesterol, Triglyceride, micro-albuminuria, urine protein and IgM index in this type of patients.

In univariate test of type1 diabetes, it is observed that age, duration of diabetes, hypertension, BMI and urine protein are associated with diabetic retinopathy. Variables with *P* < 0.2 are verified by multivariate test to determine any independent correlation with retinopathy. In multivariate test, no correlation is found between IgM index and retinopathy even after the exclusion of other variables, but a significant correlation between retinopathy and the duration of diabetes and urine protein is observed.

**Fig. 1:**
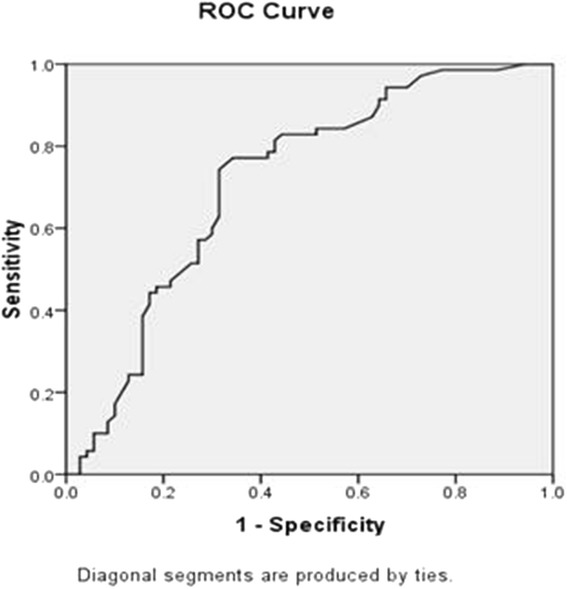
Title: Roc Curve in DM type 1. Legend: (Area under the Curve = 0.716 (CI = 0.629-0.802), *P* < 0.001)

**Fig. 2:**
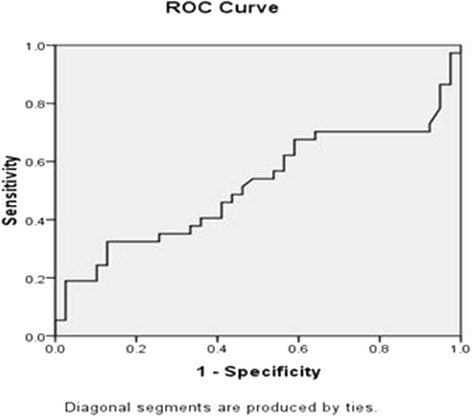
Title: Roc Curve DM type 2. Legend: (Area under the Curve = 0.517 (CI = 0.382-0.652), *P* = 0.803)

In the univariate test of type2 diabetes, it is observed that variables of ages, diabetes duration, hypertension, smoking, and FPG, HbA1c and urine creatinin are significantly correlated with retinopathy. The variables of *P*-value <0.2 are analyzed in multivariate test to verify their independent correlation with retinopathy. When other variables are excluded, the IgM index has no relationship with retinopathy while variables such as duration of diabetes and HbA1c are independently correlated with retinopathy (Table 3, Fig. 1, Fig. 2).

**Table 3:** Correlation of Variables with IgM in Type I and II Diabetic Patients (Multivariate)

	DM type I	DM type II		
Variable	OR (CI 95 %)	P-value	OR (CI 95 %)	*P*-value
Age	1.02 (0.99–1.18)	0.08	1.09 (0.99–1.19)	0.061
Sex	0.67 (0.18–2.5)	0.56	1.35 (0.4–4.60)	0.62
Duration	1.18 (1.03–1.35)	0.014	1.13 (1.03–1.23)	0.006
HTN	1.17 (0.11–12.39)	0.89	1.73 (0.68–4.40)	0.24
BMI	0.93 (0.78–1.1)	0.42	5.13 (0.63–31.57)	0.078
Cigarette	0.65 (0.60–7.17)	0.73	0.997 (0.998–1.006)	0.489
Urine Albumin	1.1 (0.20–5.91)	0.91	1.53 (1.02–2.31)	0.038
Urine Creatinin	1.003 (0.99–1.009)	0.28	1.28 (0.51–3.22)	0.59
Urine Protein	7.22 (1.68–30.95)	0.008	0.997 (0.993–1.000)	0.76
IgM Index	1.054 (0.95–1.169)	0.322	0.997 (0.983–1.01)	0.633

Based on Roc curve for type1 diabetes, multiple cut points were determined. Considering that the objective of this study was to set a criterion for screening retinopathy, and high sensitivity is desired for screening, 1.49 × 10^−3^, 0.703 and 0.308 values were defined as cut point, sensitivity and specificity respectively. Regarding type2 diabetes, determination of cut point was impossible.

## Discussion

The present study showed that the mean urinary IgM was not different between the diabetic patients with or without retinopathy, but the urinary IgM level of higher than 1.49 × 10^−3^ mg/dl could be used as a cut point for type1 diabetic patients to predict retinopathy. This study also supports the correlation of diabetes duration and urine protein with retinopathy in type1 diabetes as supported in previous studies. Besides, the correlation of diabetes duration and HbA1c with retinopathy was supported in the type2 diabetes group.

The idea of the correlation between urinary IgM and renal complications was raised long ago. In a work by Bakoush et.al [19], they found that urinary IgM excretion in type2 diabetic retinopathy is more than type1 diabetic retinopathy. Later, Bakoush et.al [20] did another study on 139 type1 diabetic patients in which they were analyzed in a follow-up test for the next 18 years. In this study, 20 patients had final-stage renal disease. This study showed that patients with different degrees of albuminuria (i.e. individuals of higher urinary IgM) final stage of renal disease are three times more common [20]. In 2012, Bakoush et.al performed a similar study on type2 diabetes patients and found that of 106 patients in a follow-up study lasting for five years, 41 individuals had final-stage renal diseases. During these analyses, it was observed that the urinary IgM is a proper predictor for renal involvement without considering the degree of albuminuria [5]. As far as the analyses of the present study are concerned, the selected patients had no significant urinary proteinuria and their values of GFR and Cr were respectively more than 90 cc (mL) and less than 1.5. Therefore, due to the limitations in the selection of patients, the investigation of the correlation between IgM and nephropathy was impossible.

During the two above-mentioned studies by Bakoush et.al, the correlation between urinary IgM and cardiovascular events was verified. In patients with type1 diabetes, the increase of urinary IgM excretion was followed by the increase of mortality due to cardiovascular events; without considering the degree of albuminuria [21]. Also, a significant correlation between micro-albuminuria and different complications of diabetes was observed. Micro-albuminuria is a risk factor for cardiovascular events in type2 diabetes [22] and it was correlated with type2 diabetes [11].

Due to the fact that vascular complications of diabetes are linked to each other, the correlation of IgM with cardiovascular events and end-stage renal disease (ESRD) was observed in diabetic patients. The analyses of the present study aimed to investigate the correlation between urinary IgM and diabetic retinopathy without considering the degree of albuminuria. The correlation between albuminuria and retinopathy was also verified in the present study. In both types of diabetes, the correlation between micro-albuminuria and diabetic retinopathy was relatively significant while this correlation was no more applied when other variables were ignored and independent correlation among variables was analyzed. This might be due to the fact that most of the patients in the present study consumed drugs such as angiotensin-converting enzyme inhibitor (ACE Inhibitors) or angiotensin receptor blocker (ARB) to control their blood pressure or albuminuria. This may be the reason for the reduction of albumin excretion in these patients. Since mechanisms of urinary IgM and albumin excretion are different, it does not seem that these mechanisms might be the confounding factor in urinary IgM excretion. In order to investigate the correlation between IgM and nephropathy, a comparison was done between the urinary IgM and the pre-determined cut point in Bakoush’s study [19]. However, no significant correlation was found. Since in that study patients suffered from advanced stages of renal diseases, a new cut point for type1 diabetes was determined which was 1.49 × 10^−3^ mg/dl. Sensitivity and specificity were 0.703 and 0.308, respectively.

The limitation of this study was the small sample size and inability to distinguish between the different degrees of retinopathy, which was one of its predefined objectives. The present study was cross-sectional but it is better to be done in cohort format. In addition, Patients with advanced types of nephropathy such as those with significant proteinuria and degrees of renal disorder were excluded from this study.

Furthermore, It is better to do the future studies in perspective form with larger samples and based on different degrees of retinopathy. More advanced cases of nephropathy should also be included along with patients who have not received treatments for blood pressure and albuminuria.

## Conclusion

The results of the present study demonstrate that in type 2 diabetic patients with and without retinopathy average values of urinary IgM was not different. However, urinary IgM level of higher than 1.49x10^-3^, could be used as a cut point to predict retinopathy in type1 diabetic patients. Similar to other studies on this subject, the present study could show long-term correlation of diabetes and proteinuria with retinopathy in type1 diabetes. It also could show the correlation of diabetes duration and HbA1c with retinopathy in type2 diabetes.

## Abbreviations

ACE Inhibitors, Angiotensin-converting enzyme inhibitor; ARB, Angiotensin receptor blocker; BMI, Body mass index; FPG, Fasting plasma glucose; HbA1c, Hemoglubin A1c; HTN, Hypertention; IgM, Immunoglubulin M
